# Clinical characteristics and lateralization of the horizontal semicircular canal light cupula

**DOI:** 10.3389/fneur.2024.1357195

**Published:** 2024-02-21

**Authors:** Wenjing Qin, Zheng Liu, Yanhan Zhu, Xueyan Zhang, Jiao Xu, Tao Zhou, Lingli Wei, Yi Fang, Liying Chang

**Affiliations:** Department of Neurology, Xiangyang Central Hospital, Affiliated Hospital of Hubei University of Arts and Science, Xiangyang, China

**Keywords:** light cupula, direction-changing positional nystagmus, null point, supine roll test, bow and lean test, caloric test

## Abstract

**Introduction:**

Positional vertigo and nystagmus are the main symptoms and signs of dizziness, respectively. Despite the clinical utility of the supine roll test (SRT) and null point (NP) in diagnosing light cupula, a type of positional vertigo, there exists a notable gap in the literature concerning the comprehensive evaluation of lateralization values based on various nystagmus characteristics and the intensity of direction-changing positional nystagmus (DCPN) in the SRT, particularly in comparison to the NP. Additionally, limited data on abnormal canal paresis (CP) in light cupula patients underscores the need for further research with a larger patient population to elucidate this mechanism. This study aims to investigate the characteristics of positional nystagmus and lateralization of the horizontal semicircular canal (HSCC) light cupula, which is a type of positional vertigo and nystagmus that is poorly understood.

**Methods:**

Eighty-five patients (17 males, 68 females; mean age, 60.9 years) with light cupula were reviewed. We summarized the characteristics of spontaneous nystagmus and positional nystagmus, including supine positioning nystagmus, bow nystagmus, and lean nystagmus. Then, the side of the NP was identified as the affected side, and the values of the fast phase direction of the spontaneous nystagmus, supine positioning nystagmus, bow nystagmus, and lean nystagmus, as well as the intensity of the DCPN in the SRT, were used to diagnose the affected sides. Caloric testing was also performed for some patients.

**Results:**

Light cupula was observed in 5.7% of the patients with positional nystagmus. The frequencies of supine positioning nystagmus (88.2%), bow nystagmus (90.6%), and lean nystagmus (83.5%) were higher than spontaneous nystagmus (61.2%) (*p* < 0.001). The second NP (NP2) (92.9%) and third NP (NP3) (83.5%) were readily detected, affecting the left and right sides in 38 and 47 patients, respectively. Lateralization through the fast phase directions of bow nystagmus and lean nystagmus did not significantly differ from that of NP (all *p* > 0.05). However, the accuracy rate of lateralization through the sides with more vigorous DCPN in the SRT was 63.5%, significantly lower than through NP (*p <* 0.001). Particularly in patients with supine positioning nystagmus (*n* = 75), the rate was only 58.7% (*p <* 0.001). However, the rate was 100% in patients without supine positioning nystagmus (*n* = 10). Among the 70 patients who underwent caloric testing, 37 had abnormal CP, and the sides of the reduced caloric reaction were ipsilateral to the affected sides of the light cupula in 83.8% of the patients.

**Conclusion:**

Besides utilizing the NP to determine the affected side, the fast phase direction of the bow nystagmus or lean nystagmus can also aid in identification. However, a simple comparison of the intensity of DCPN in SRT cannot provide accurate lateralization, especially in patients with supine positioning nystagmus. There is a high incidence of CP on the affected side of the light cupula.

## 1 Introduction

Positional vertigo and nystagmus are the main symptoms and signs of dizziness, respectively. Benign paroxysmal positional vertigo (BPPV), with a lifetime prevalence of 2.4%, accounts for approximately 80% of all cases of positional nystagmus (1, 2). Generally, transient direction-changing positional nystagmus (DCPN) with a latency of a few seconds is characteristic of horizontal semicircular canal (HSCC) canalolithiasis (3). However, persistent DCPN without latency has been observed and proven to be different (4–6). In 2001, Alexandre et al. found the disappearance point of the DCPN when rotating the head left or right from the supine position at approximately 10–20° in these patients and called it the null point (NP) (5). In 2002, Shigeno et al. proposed the term “light cupula” to explain the phenomenon of persistent geotropic DCPN (6).

Subsequently, several studies have reported the characteristics of light cupula (4, 7–14). Spontaneous nystagmus is more prevalent in light cupula cases than canalolithiasis. Moreover, spontaneous nystagmus is believed to stem from the upward deflection of the cupula, causing it to beat toward the unaffected side (8, 15). In the bow and lean test (BLT), 83–100% of patients with light cupula present with bow nystagmus and/or lean nystagmus (4, 9, 16). The fast phase direction of bow nystagmus corresponds to the affected side of the light cupula (4, 9). However, lean nystagmus is the opposite of bow nystagmus (16, 17). Additionally, the evoked nystagmus in the supine position occurs in 80–90% of patients (9, 18). Most supine positioning nystagmus are directed toward the unaffected side (9, 11, 18).

Studies have shown that the intensity of the DCPN in the supine roll test (SRT) helps determine the affected side using Ewald’s second law (4, 9, 15). It is typically used clinically. Moreover, the NP is gradually being studied as an important and typical characteristic of light cupula, and the side of the NP is credibly considered the affected side (4, 11, 15, 19, 20). All these features help make a diagnosis and identify the affected side of the light cupula (4, 7, 17). However, the values of lateralization by the fast phase directions of the spontaneous nystagmus, supine positioning nystagmus, bow nystagmus, or lean nystagmus or by the intensity of DCPN in the SRT, as compared to the NP, have been poorly evaluated. Recently, three studies reported abnormal canal paresis (CP) in patients with light cupula (9, 18, 21). However, the number of patients included in these studies was small. More data are required to clarify this mechanism further.

In this study, the cases of 85 patients with HSCC light cupula were reviewed, and the characteristics of nystagmus (spontaneous nystagmus, supine positioning nystagmus, bow nystagmus, lean nystagmus, and DCPN in the SRT), NP, and caloric tests were investigated. Spontaneous nystagmus, supine positioning nystagmus, bow nystagmus, lean nystagmus, and SRT values for lateralization were also analyzed.

## 2 Materials and methods

### 2.1 Patient selection

We conducted a retrospective study of 85 patients with light cupula from 1,485 patients with positional vertigo and nystagmus who were admitted to the Department of Neurology, Xiangyang Central Hospital, Affiliated Hospital of Hubei University of Arts and Science between January 2021 and December 2022. The diagnostic criteria for light cupula were as follows: (1) Persistent geotropic DCPN lasting for more than 1 min without latency was recorded during SRT; (2) The NP was identified; and (3) There were no central nervous system lesions (13). All patients underwent SRT and the Dix–Hallpike test (DHT). A thorough neurological examination, including measurement of consciousness level, language function, pupil size, eye movement, gaze-evoked nystagmus, facial muscle movement, limb muscle strength, pathological reflex, balance assessment (alternating movement test, finger-to-nose test, and heel-knee-tibia test), and brain magnetic resonance imaging, were performed to confirm the absence of central nervous system lesions. This study was approved by our hospital’s Ethics Committee (2022-069).

### 2.2 Test procedures

Nystagmus was recorded using a videonystagmography (VNG) system (Interacoustics, Assens, Denmark). The VNG system automatically recorded the maximum slow-phase velocity (SPV) of the 10 s strongest nystagmus.

First, the patient was placed in a sitting position without head movement to assess spontaneous nystagmus (22). Next, the patient was placed in the supine position with the head bent forward by approximately 30° to detect the supine positioning nystagmus (18). In the SRT, the head was rapidly rotated 90° to one side, returned to the supine position, and rotated 90° to the other side (3). In the DHT, the patient is placed in a sitting position with the head rotated 45° to one side and then rapidly laid down in a head-hanging position (3). The patient was returned to the sitting position, and the procedure was performed on the other side similarly. In the BLT, the head of the sitting patient is bent forward at 90° to detect the bow nystagmus and then tilted backward at 60° to detect the lean nystagmus (16). During testing, the presence or absence of nystagmus was observed at every position, and if nystagmus appeared, the duration, SPV, and fast phase direction were recorded. The patient was maintained in every position for 2 min to achieve nystagmus remission.

Subsequently, three NPs were identified (15). First, the head of the patient who showed spontaneous nystagmus was slightly bent forward at an angle where the nystagmus ceased; this point was called the first NP (NP1). The patient was then placed in the supine position with the head bent forward at 30°, and the head was rotated left or right at an angle at which the nystagmus ceased; this point was the second NP (NP2). Finally, the head was bowed at 90° and rotated left or right at an angle at which nystagmus ceased; this point was the third NP (NP3).

For the caloric test, the patient was placed in a supine position with the head bent forward at 30° (23). The right and left ears were then, respectively, irrigated with air at 24°C or water at 30°C, followed by irrigation with air at 50°C or water at 44°C. The stimulation lasted 1 min with 10 L/min airflow or 30 s with 250 mL of water. The CP values were calculated using the Jongkees formula. Unilateral hypofunction was defined as CP >25%. Bilateral hypofunction was confirmed when the sum of the ipsilateral SPV of caloric-induced nystagmus by cold and warm stimulation was <6°/s on both sides (23).

### 2.3 Data collections

Demographic data (sex and age) of the 85 patients were collected. Data on nystagmus (the SPV and the fast phase directions of the spontaneous nystagmus, supine positioning nystagmus, bow nystagmus, lean nystagmus, and DCPN in the SRT), NP (the angles of NP1, NP2 and NP3, and the affected sides), and caloric test (CP values and the hypofunctional sides) were collected. Referring to the NP standard, the accuracy of lateralization was assessed by observing the fast phase directions of the spontaneous nystagmus, supine positioning nystagmus, bow nystagmus, and lean nystagmus, as well as the intensity of DCPN in the SRT.

### 2.4 Statistical analysis

The quantitative data, including ages and CP values, which followed a normal distribution, were presented as mean ± standard deviation values. A comparison of ages between males and females was conducted using an independent-sample *t-*test. Conversely, quantitative data, such as the SPV of nystagmus that did not conform to a normal distribution, were presented as the median (lower quartile, upper quartile). In the SRT, the SPV of nystagmus on the affected and unaffected sides was compared using the nonparametric Wilcoxon test. All remaining count data were presented as percentages, and some of the data to be compared were analyzed using Pearson’s Chi-square test; if necessary, Fisher’s exact test was used. All statistical tests were two-sided, and a significance level of *p* < 0.05 was considered statistically significant. The analysis was performed using the SPSS software package (version 22.0; IBM Corp., Armonk, NY, United States).

## 3 Results

### 3.1 Clinical baseline characteristics

Eighty-five patients diagnosed with light cupula involving the HSCC were included (Table 1). They were 17 males and 68 females, with an average age of 60.9 ± 11.9 years. Females were more common than males. There were no significant differences in the ages of males (61.8 ± 13.2 years) and females (60.6 ± 11.7 years) (*p* = 0.707).

**Table 1:** Clinical characteristics of patients with light cupula.

Total number of cases (*n*)	85
Age of onset, mean ± SD (*y*, range)	60.9 ± 11.9 (14–81)
Sex ratio: female/male (*n*)	68/17
Incidence of nystagmus (*n*, percent)	
Spontaneous nystagmus	52 (61.2%)
Supine positioning nystagmus	75 (88.2%)
Bow nystagmus	77 (90.6%)
Lean nystagmus	71 (83.5%)
NP (*n*, percent)	
NP1	42 (49.4%)
NP2	74 (87.1%)
NP3	70 (82.4%)
Affected side: right/left (*n*)	47/38
Caloric test (*n*)	70
The abnormal CP (*n*, percent)	37 (52.9%)

NP, null point; NP1, the first NP; NP2, the second NP; NP3, the third NP; CP, canal paresis.

### 3.2 Spontaneous and positional nystagmus characteristics

In patients with HSCC light cupula, 61.2% presented with spontaneous nystagmus with an SPV of 3 (3, 5)°/s, 88.2% showed supine positioning nystagmus with an SPV of 4 (3, 8)°/s, 90.6% showed bow nystagmus with an SPV of 7 (3.5, 12)°/s, and 83.5% showed lean nystagmus with an SPV of 6 (3, 10)°/s. There were significant differences in the frequencies of the spontaneous nystagmus, supine positioning nystagmus, bow nystagmus and lean nystagmus (*p <* 0.001). The spontaneous nystagmus was rarer than the supine positioning nystagmus, bow nystagmus, and lean nystagmus (all *p* < 0.05).

### 3.3 NP

The NP1 was observed in 49.4% of the patients with angles measuring 20° (15°, 30°). NP2 was observed in 87.1% of the patients with angles measuring 26.5° (20°, 30°). NP3 was observed in 82.4% of the patients with angles of 20° (15°, 27°). As NP2 and/or NP3 were observed in all patients, the affected sides of 44.7% were on the left, and 55.3% were on the right, according to the sides of the NP.

### 3.4 Caloric test

Among the 70 patients assessed in the caloric test, 52.9% exhibited abnormal CP values, indicating unilateral vestibular hypofunction, while 47.1% showed normal CP values. None of the patients had bilateral vestibular hypofunction. The mean abnormal CP values were 44.8 ± 15.9%, ranging from 26 to 89%, and the mean normal CP values were 8.4 ± 5.7%, ranging from 0 to 20%. In 83.8% of patients, the sides of the abnormal CP were ipsilateral to the sides of the NP.

### 3.5 Lateralization diagnosis

By identifying the sides of the NP as the affected sides, the fast phase directions were as follows: 90.4% of the spontaneous nystagmus, 93.3% of the supine positioning nystagmus, and 95.8% of the lean nystagmus were toward the unaffected sides, while 97.4% of the bow nystagmus were toward the affected sides. Significant differences were observed in the accuracies of lateralization through the fast phase directions of the spontaneous nystagmus, or supine positioning nystagmus compared with NP (*p* = 0.007 and *p* = 0.021, respectively). However, no significant differences were observed in the accuracies of lateralization through the fast phase directions of bow nystagmus or lean nystagmus compared with NP (all *p* > 0.05) (Table 2).

**Table 2:** Accuracies of lateralization as compared with NP (%).

Classification categories		Correct rate	Error^a^ rate	*p-*value
By the fast phase directions	Spontaneous nystagmus	90.4%	9.6%	0.007
	Supine positioning nystagmus	93.3%	6.7%	0.021
	Bow nystagmus	97.4%	2.6%	0.224
	Lean nystagmus	95.8%	4.2%	0.092
By the more vigorous DCPN in the SRT	without supine positioning nystagmus	100%	0%	–
	with supine positioning nystagmus	58.7%	41.3%	<0.001

a The errors included incorrect and difficult lateralization.

In the SRT, the SPV of DCPN on the affected side was 13 (8, 20)°/s, ranging from 2 to 61°/s, while on the unaffected side, it was 7 (5, 12.5)°/s, ranging from 1 to 26°/s. The intensity of DCPN on the affected side was significantly higher than on the unaffected side (*p <* 0.001). However, the accuracy rate of lateralization, based on the side with stronger DCPN during the SRT, was only 63.5%. This rate was significantly lower than NP (*p <* 0.001). Specifically, the sides with more vigorous nystagmus in the SRT were the affected sides in all 10 patients (100%) without supine positioning nystagmus and in only 44 of 75 patients (58.7%) with supine positioning nystagmus (Table 2).

## 4 Discussion

SRT-induced DCPN accounts for approximately 14–22% of all BPPV (24, 25). Transient geotropic or apogeotropic DCPN is the most prevalent type and the typical characteristic of canalolithiasis (3, 13, 14). The mechanism is well-defined, explaining the movement of the endolymph due to the displacement of free-floating otoconia (3). Furthermore, persistent apogeotropic DCPN demonstrates cupulolithiasis, also known as heavy cupula (13, 26). This phenomenon results from the dislodged otoconia adhering to the cupula (20, 26). Persistent geotropic DCPN is identified as a characteristic of light cupula (7). Previous studies have posited that cupula deflection is induced by the imbalance of specific gravity between the cupula and endolymph (4, 20). Mechanism has been explained as the “heavier endolymph hypothesis” with increased endolymph specific gravity due to an acute attack such as labyrinth hemorrhage, or inflammation in the inner ear; “lighter cupula hypothesis” based on alcohol making the cupula lighter than endolymph; “light particle hypothesis” due to the buoyancy of light debris adhering to the cupula and the “altered endolymph/perilymph density ratio hypothesis” (27). However, the exact underlying mechanisms remain unclear which cause difficulties in guiding the treatment. It is clear that the canalith-repositioning maneuver is not effective (27). In this study, light cupula was identified in 5.7% of the patients who exhibited positional nystagmus, with a male-to-female ratio of 1:4. Ichijo et al. reported a ratio of 1:2.3, while Kim et al. reported 1:2.8 (19, 24). Similar to BPPV, females appear to exhibit a higher susceptibility to the development of light cupula than males (27–29).

In this study, supine positioning nystagmus, bow nystagmus, and lean nystagmus were more common than spontaneous nystagmus, consistent with previous findings (8, 9, 16, 18). Subsequent analysis revealed no statistically significant differences in the accuracy of lateralization when testing the fast phase directions of bow nystagmus or lean nystagmus compared with NP. These results indicate that accurate lateralization can be achieved using the BLT, making it a convenient method for clinical use (27).

**Figure 1:**
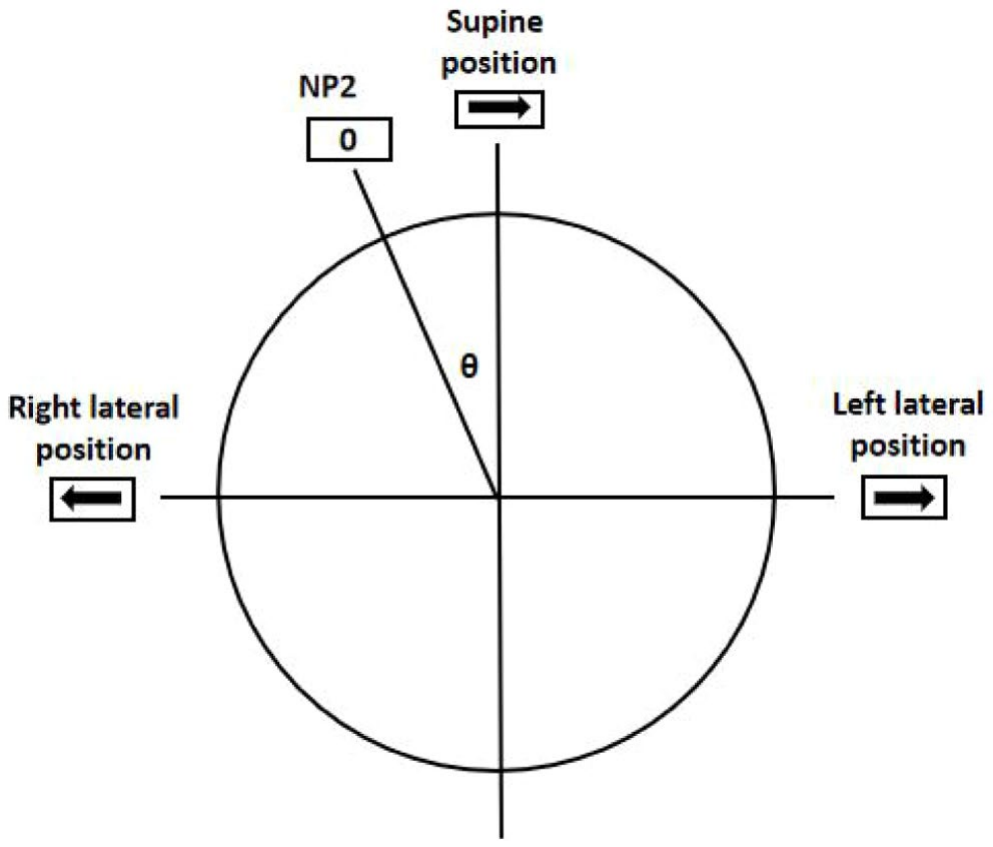
Geotropic DCPN in the SRT. The excitatory discharge was due to the cupula deflection at an angle of (90°-θ) when rotated 90° to the affected side from the supine position. The inhibitory discharge was due to the cupula deflection at an angle of (90°+θ) when rotated 90° to the unaffected side from the supine position. θ was the degree of the NP2; 0 was the point of nystagmus disappearing; → and ← showed the fast phase directions of nystagmus.

According to Ewald’s second law, the intensity of the nystagmus on the side of direct excitation (ipsilateral excitatory discharge when oblique toward the ampulla) is stronger than that of relative excitation (contralateral excitatory discharge when oblique away from the ampulla) (13, 30). This was verified in the SRT in this study. However, when directly inferring the affected sides according to the sides with stronger DCPN, the accuracy rate of lateralization was only 63.5%, which was statistically lower than that of NP. This rate is similar to those reported in previous studies (Kim, 68%; Seo, 67%) (4, 31). Furthermore, in a recent study, only 16% was reported (32). This indicates that it is difficult to determine the affected side by comparing the intensity of the DCPN during the SRT (27). The sides with stronger nystagmus were the affected sides in all 10 patients without supine positioning nystagmus but in only 44 of 75 patients with supine positioning nystagmus. We found that in patients with supine positioning nystagmus, when the head is rotated 90° toward the affected side from the supine position, the excitatory discharge must first offset the inhibitory discharge caused in the supine position. The excitatory discharge on the affected side resulted from the cupula deflection at an angle of 90°-θ (where θ represents the degree of NP2) (Figure 1) (32).

Conversely, when the head rotated 90° toward the unaffected side, an inhibitory discharge was added to that caused in the supine position. Consequently, the inhibitory discharge on the unaffected side was due to the cupula deflection at an angle of (90° + θ) (32). Hence, a simple comparison of nystagmus intensity in the SRT cannot be employed to identify affected sides in patients with supine positioning nystagmus due to the different degrees of cupula deflection and neural discharge patterns. However, such comparisons can be performed in patients without supine positioning nystagmus to determine the affected side.

In the caloric test, 52.9% of the patients had abnormal CP. The sides with reduced caloric response were ipsilateral to the affected side of the light cupula in 83.8% of the patients. Consequently, approximately 44% of patients experienced CP on the lesion side. Tomanovic et al. reported a pathologic caloric ratio (≥20%) in 65% of 20 patients (18). Ichijo found that 21% of 14 patients had an abnormal CP, and the reduced sides of all were on the affected sides (21). Si et al. reported abnormal CP in 43.3% of 30 patients, with 61.5% having reduced reaction on the affected sides (9). Therefore, a high incidence of CP on the affected side is evident in patients with light cupula. It may result from morphological changes of the cupula as well as from endolymphatic disorders that change the morphology of the cupula (27). We assumed that it was closely associated with the light cupula. Further research is required to clarify the underlying mechanisms.

## 5 Conclusion

Light cupula is not rare, and females are more susceptible. Evaluating the NP at which the nystagmus disappears is essential for diagnosis and lateralization. Observing the fast-phase directions of the bow nystagmus or lean nystagmus helps diagnose the affected sides. However, identifying the sides with a stronger DCPN in SRT cannot achieve accurate lateralization, especially in patients with supine positioning nystagmus. Moreover, 44% of patients with light cupula experience ipsilateral abnormal caloric response.

## 6 Study limitations

The study lacked data on patients’ medical history and comorbid disorders, such as vestibular migraine, Meniere’s disease, and sudden sensorineural hearing loss, which would be useful in analyzing the etiology. And the study lacked data on the video head impulse test (vHIT) and the vestibular evoked myogenic potential (VEMP), maybe making the result of vestibular dysfunction underestimate. The single-centrer study might restrict the ability to identify significant effects. Further studies are needed to explore the characteristics and mechanisms.

## Data availability statement

The original contributions presented in the study are included in the article/supplementary material, further inquiries can be directed to the corresponding authors.

## Ethics statement

The studies involving humans were approved by the Ethics Committee of Xiangyang Central Hospital, Affiliated Hospital of Hubei University of Arts and Science (2022-069). The studies were conducted in accordance with the local legislation and institutional requirements. The participants provided their written informed consent to participate in this study.

## Author contributions

WQ: Data curation, Formal analysis, Supervision, Writing – original draft. ZL: Data curation, Formal analysis, Investigation, Writing – review & editing. YZ: Methodology, Resources, Software, Writing – review & editing. XZ: Data curation, Methodology, Resources, Writing – review & editing. JX: Resources, Supervision, Writing – review & editing. TZ: Data curation, Resources, Writing – review & editing. LW: Resources, Software, Writing – review & editing. YF: Conceptualization, Supervision, Visualization, Writing – review & editing. LC: Conceptualization, Supervision, Visualization, Writing – review & editing.
